# Modeling gene expression regulatory networks with the sparse vector autoregressive model

**DOI:** 10.1186/1752-0509-1-39

**Published:** 2007-08-30

**Authors:** André Fujita, João R Sato, Humberto M Garay-Malpartida, Rui Yamaguchi, Satoru Miyano, Mari C Sogayar, Carlos E Ferreira

**Affiliations:** 1 Institute of Mathematics and Statistics, University of São Paulo, Rua do Matão, 1010 – São Paulo, 05508-090, SP, Brazil; 2 Chemistry Institute, University of São Paulo, Av. Lineu Prestes, 748 – São Paulo, 05513-970, SP, Brazil; 3 School of Arts, Science and Humanities, University of São Paulo, Av. Arlindo Bettio, 1000 – São Paulo, 03828-000, SP, Brazil; 4 Human Genome Center, Institute of Medical Science, University of Tokyo, 4-6-1 Shirokanedai, Minato-ku, Tokyo, 108-8639, Japan

## Abstract

**Background:**

To understand the molecular mechanisms underlying important biological processes, a detailed description of the gene products networks involved is required. In order to define and understand such molecular networks, some statistical methods are proposed in the literature to estimate gene regulatory networks from time-series microarray data. However, several problems still need to be overcome. Firstly, information flow need to be inferred, in addition to the correlation between genes. Secondly, we usually try to identify large networks from a large number of genes (parameters) originating from a smaller number of microarray experiments (samples). Due to this situation, which is rather frequent in Bioinformatics, it is difficult to perform statistical tests using methods that model large gene-gene networks. In addition, most of the models are based on dimension reduction using clustering techniques, therefore, the resulting network is not a gene-gene network but a module-module network. Here, we present the Sparse Vector Autoregressive model as a solution to these problems.

**Results:**

We have applied the Sparse Vector Autoregressive model to estimate gene regulatory networks based on gene expression profiles obtained from time-series microarray experiments. Through extensive simulations, by applying the SVAR method to artificial regulatory networks, we show that SVAR can infer true positive edges even under conditions in which the number of samples is smaller than the number of genes. Moreover, it is possible to control for false positives, a significant advantage when compared to other methods described in the literature, which are based on ranks or score functions. By applying SVAR to actual HeLa cell cycle gene expression data, we were able to identify well known transcription factor targets.

**Conclusion:**

The proposed SVAR method is able to model gene regulatory networks in frequent situations in which the number of samples is lower than the number of genes, making it possible to naturally infer partial Granger causalities without any *a priori *information. In addition, we present a statistical test to control the false discovery rate, which was not previously possible using other gene regulatory network models.

## Background

In order to understand cell functioning as a whole, it is necessary to describe, at the molecular level, how gene products interact with each other. This could help to identify new target genes and to design new drugs for treatment of several diseases [1-3]. Due to the high number of genes involved in these networks, activating or suppressing feedback loops, the dynamics of their interactions is very complex and difficult to infer.

With the development of high-throughput technologies, such as DNA microarrays, it is possible to simultaneously analyze the expression of up to thousands of genes and to construct gene networks based on inferences over gene expression data.

Several methods to model genetic networks were proposed in the last few years, such as the Bayesian networks [4-8], Structural Equation Models [9], Probabilistic Boolean Networks [10-12], Graphical Gaussian Models [13], Fuzzy controls [14], and Differential Equations [15].

Although these methods allow modeling several regulatory networks for which biological information is available, it is difficult to determine the flow of information when there is no *a priori *knowledge.

In addition, all of these methods face the same problem, i.e., the number of samples (microarrays) is very small, when compared to the high number of variables (genes) (ill posed problems, related to the "curse of dimensionality") [16]. Therefore, it is difficult to infer large scale networks using traditional statistical methods, limiting this inference to only a few genes. As a consequence, modeling and simulating large networks becomes a field of intensive and challenging research. At this point, it is important to define what is considered a "large" network. We consider as "large" a network in which the number of genes is larger than the number of microarrays experiments, implying in a large number of parameters to be estimated.

Some methods have been developed to overcome this problem. For example, Barrera *et al*. use mutual information for dimension reduction [17], with mutual information between genes being computed and then, the highest mutual informations selected. However, this approach is not founded on a statistical test, rendering it very difficult to interpret and identify the actual edges of the network. Therefore, the choice of the threshold parameter to determine whether there is or not a connection, becomes quite subjective. An alternative to model the large number of genes is to construct modules (clusters), where each module is composed by several genes, and then, to construct the module-module networks [18-20]. A limitation of these methods is that they still are not a gene-gene network, therefore, interpretation of the meaning of each module is difficult, varying with each cluster.

Here we present the Sparse Vector Autoregressive model to approach these problems. This method was first applied, with success, in neurosciences, to estimate functional connectivity between several brain areas [21]. Here, we present the Sparse Vector Autoregressive model based on LASSO penalized regression for variable selection to reduce the dimensionality on large gene networks.

In cases of multiple time series, a first approach to infer connectivity would be to apply techniques such as multivariate autoregressive modeling (VAR), which allows identification of connectivity by combining graphical modeling methods with the concept of Granger causality [22]. This is an attractive approach since it does not require *a priori *network information. Unfortunately, the current time series methods can only be applied only for cases in which the length of the time-series *T *is much larger than *n*, the number of genes, which is exactly the reverse of the situation commonly found in microarray experiments, for which relatively short time-series are measured over tens of thousands of genes. The Sparse Vector AutoRegressive model (SVAR), on the other hand, estimates the network in a two-stage process involving (i) penalized regression with LASSO regression [23] and (ii) pruning of unlikely connections by means of the False Discovery Rate (FDR) developed by [24]. Extensive simulations were performed with artificial gene networks having scale-free like topologies [25] and stable dynamics. These simulations show that the detection efficiency of connections of the proposed procedure is quite high. An application of the method to actual HeLa cell line data was illustrated by the identification of well known transcription factor targets and circuitries involving important genes in cancer development.

## Results and discussion

In order to measure the performance of SVAR, intensive simulations were carried out. For this purpose, we simulated hundreds of networks with scale-free like topology since the metabolic network was described as scale-free graphs by [25]. In our case, the graph nodes represent the genes whereas the edges represent the Granger-causal relationships. For details of these artificial regulatory networks, see the Methods section.

**Figure 1:**
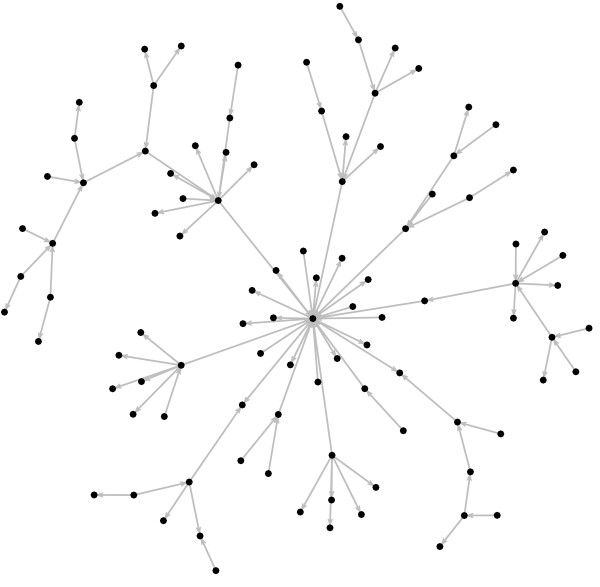
**Artificial gene regulatory network**. Example of a simulated sparse gene regulatory network with *n *= 100 genes and 100 connections. The arrows indicate the Granger-causal relationships.

The number of genes was kept at *n *= 100 and we varied the sample size, i.e., the time-series length (time-series length *T *= 25, 50, 75, 100, 125, 150, 175 and 200 for SVAR and *T *= 110, 125, 150, 175 and 200 for VAR). Notice that, for VAR of order one, *m *= *T *- 1 must be larger than *n*. For each time-series length, we performed 100 simulations, i.e., 100 different scale-free like graphs were generated. The starting conditions of the scale-free like graphs were two fully connected genes (*z*_0 _= 2, *z*_*edges *_= 2, where *z*_0 _is the initial number of genes and *z*_*edges *_is the initial number of edges), in other words, two nodes with two edges, one pointing to the other. The number of edges added at each iteration is *z *= 1, therefore, each network is composed by 100 genes and 100 edges out of 10,000 possible edges (the maximum number of possible edges is *n*^2^). Notice that since the goal is to construct a network with *n *= 100 genes, we set the number of iterations *T*_*step *_= *n *- *z*_0 _= 98. In Figure 1, an example of the artificially generated gene expression regulatory network is illustrated.

**Figure 2:**
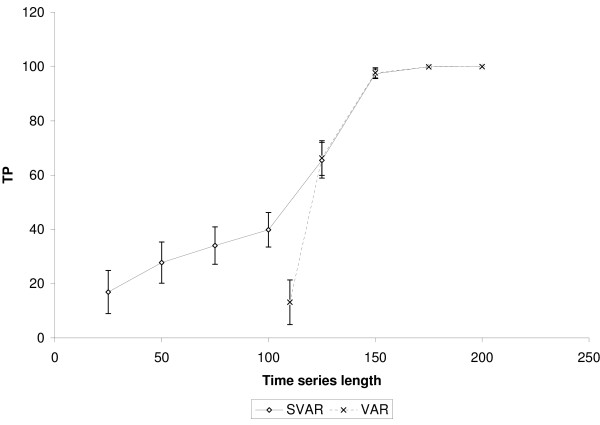
**Comparison between SVAR and VAR**. The simulations were performed in a scale-free like network composed of 100 nodes and 100 edges. VAR was performed only for experiments with the length of the time-series of up to 110. TP: True positives. The number of false positives is controlled using q-value < 0.01. The error bar is representing one standard error.

**Figure 3:**
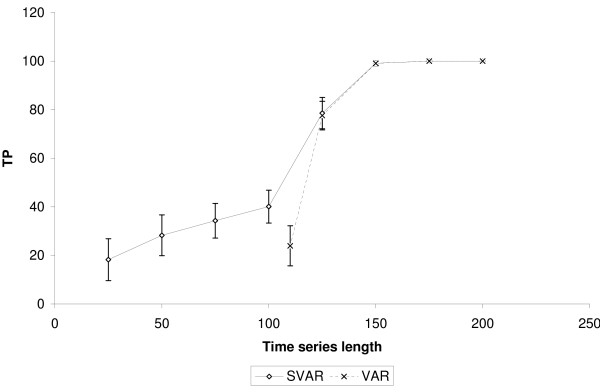
**Comparison between SVAR and VAR**. The simulations were performed in a scale-free like network composed of 100 nodes and 100 edges. VAR was performed only for experiments with the length of the time-series of up to 110. TP: True positives. The number of false positives is controlled using q-value < 0.05. The error bar is representing one standard error.

**Figure 4:**
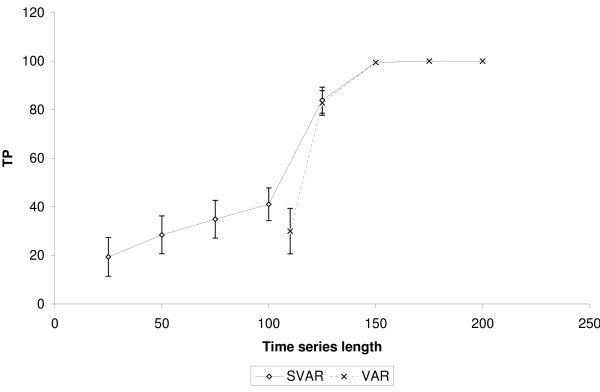
**Comparison between SVAR and VAR**. The simulations were performed in a scale-free like network composed of 100 nodes and 100 edges. VAR was performed only for experiments with the length of the time-series of up to 110. TP: True positives. The number of false positives is controlled using q-value < 0.10. The error bar is representing one standard error.

It is important to highlight that SVAR was able to identify true positive edges even when the time-series length was lower than the number of genes. Figures 2, 3 and 4 show, respectively, the number of true positives inferred by SVAR and VAR for controlled false positives rate, i.e., q-value (error type I rate within rejected hypotheses) thresholds lower than 0.01, 0.05 and 0.10. Since the estimated *β*'s standard error is proportional to the time series' length (the greater the time series, the lower is the *β*'s standard error) we varied only the time series' length.

Analyzing figures 2, 3 and 4, we obtained the following results:

1. The capacity of SVAR to identify true positives even when the number of samples is lower than the number of genes is satisfactory. This was found when comparing the performance between SVAR, with the time-series length equal to 50, and VAR, with time-series length equal to 110. Also, in this case, SVAR has identified more true positive edges than VAR (the proportion of the quantity of true positives inferred by SVAR is about 75% higher than the number of true positives inferred by VAR).

2. By comparing SVAR and VAR when the number of genes is lower than the number of samples, in general, SVAR is slightly more powerful than VAR, since the number of connectivities is larger than the number of samples.

3. When *m *≫ *n*, where *m *= *T *- 1 and *n *is the number of genes, there is no statistical difference between SVAR and VAR. This could be explained, in this context, because the best *λ *which minimizes the GCV (Generalized Cross-Validation) is near to zero. When *λ *= 0, the SVAR model becomes the traditional VAR model.

**Figure 5:**
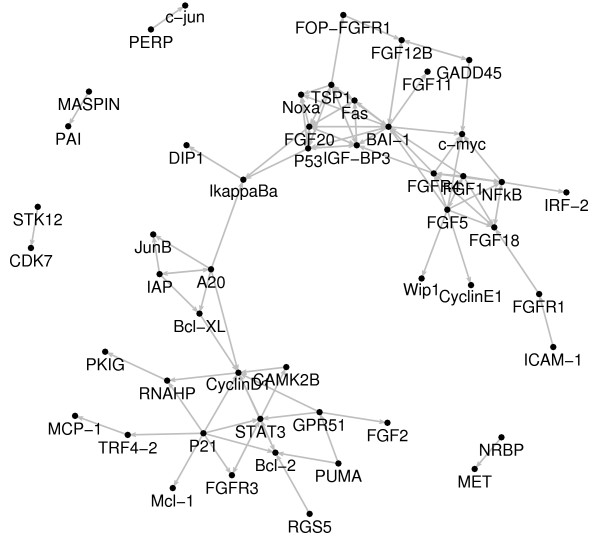
**HeLa gene expression regulatory network**. Gene regulatory network inferred from HeLa cell cycle gene expression data. The arrows represent the Granger-causal associations with q-value < 0.05. Genes with no Granger-causal links identified by SVAR were not plotted.

We have also analyzed the expression profile of a set of 94 cell cycle-regulating genes represented by 48 microarrays, i.e., the number of genes *n *is approximately 2 times larger than the time-series length *T*. Figure 5 shows the genes that display any connectivity under a false-positive rate (FDR) of 5% (q-value < 0.05). Genes with no connectivity were excluded.

The SVAR method reveals at least three gene regulatory networks related to cell transformation and tumor progression, namely: NF*κ*B, p53, and STAT3 transcriptional modules [26-28], which is in agreement with already well known cell cycle-regulated pathways in several cellular models and in Hela cells themselves.

It is important to highlight that the out-degree (number of edges with the gene as their initial vertex) of genes encoding proteins that act as well-known transcriptional factors (p53, NF*κ*B and STAT3) or important genes for cell proliferation control (*p21*, *bai1*, *tsp1*, *a20*) is higher than that of other genes. In a similar analysis, the in-degree (number of edges with the gene as their terminal vertex) of the FGFs (*fgf18*, *fgf20*, *fgfr4*) and of genes involved in cell cycle regulation and apoptosis (*cyclin d1*, *c-myc*, *bcl-2*, *noxa*, *fas*) is also higher, demonstrating the association between their key role in cell homeostasis and their in-degree and/or out-degree values [29].

NF*κ*B is an inducible transcription factor complex formed by heterodimeric association between *relA *and *c-rel *gene products, whose transcriptional activity is regulated by interaction with the inhibitory I*κ*B*α *protein. It has already been demonstrated that activation of NF*κ*B controls cell-cycle progression in HeLa cells by several mechanisms [30]. The SVAR method was not able to identify the relationship between NF*κ*B and its natural targets, such as *A20*, *iap*, *bclx *and *iκBα *genes. However, SVAR is showing that NF*κ*B directly regulates several fibroblast growth factors (FGFs) and the c-Myc protein, which are key regulators of cell proliferation. Indeed, it is noticed that the majority of NF*κ*B transcriptional activity is mediated by interaction with FGFs-related proteins, at the upstream and/or downstream levels. These results support the hypothesis that some of the multiple aspects of tumorigenesis in Hela cells may be related to NF*κ*B -mediated transcription of FGFs-related proteins.

As discussed above, the positive NF*κ*B regulation of several well-known natural targets was not detected by SVAR. However, these regulatory processes appear to be present, even in the absence of an evident direct link with NF*κ*B, since all of these transcriptionally regulated genes form a highly related network (Figure 5). A20, a zinc finger protein, which is transcriptionally regulated by NF*κ*B in several cell types [31], appears to orchestrate the genes relationship in this network, activating the transcription of well-known anti-apoptotic genes, such as *iap*, *bclx *and *junB *– NF*κ*B target genes themselves [32-34] – towards transduction of the proliferative transcriptional activity of NF*κ*B. The A20 protein is also involved in NF*κ*B regulation, blocking its activity, in a negative feedback mechanism [35]. Although this control is operated at the post-transcriptional level, results obtained using the SVAR method suggest that this process could also be controlled by A20-mediated positive regulation of *iκBα *(Figure 5). These results confirm the reliability of SVAR for predicting gene relationship, since *iκBα*, the natural NF*κ*B inhibitor has a key role in controlling the NF*κ*B -regulated cell cycle events in Hela cells, as referred to in literature [30]. Moreover, SVAR showed that this role of *iκBα *in Hela cell cycle progression also appears to be regulated through p53-mediated activation of *iκBα *(Figure 5), in agreement with data reported in the literature [36]. In summary, these data support the hypothesis that *iκBα *may be involved in attenuation of tumor progression and be responsible for the mildly invasive phenotype displayed by Hela cells.

The p53 protein is a transcription factor that binds to the enhancer/promoter elements of downstream target genes and regulating their transcription and initiating cellular programs that account for most of its tumor-suppressor functions, namely: cell cycle arrest, inhibition of angiogenesis and metastasis, apoptosis induction and DNA repair [37]. The SVAR method was capable of identifying the interactions of several members of the p53 network. IGF-BP3 (IGF-binding protein 3), an inhibitor of insulin-like growth factor, and NOXA, a BCL-2 homology domain 3-only (BH3-only) protein, are transcriptionally activated by p53 in activation of apoptosis in several cell types [38,39]. Our *in silico *results showed that this regulation is also present in Hela cells. Although the *fas *gene is not a known target of p53, its activation could be mediated by other p53 targets, leading to increased apoptosis rate and cell proliferation control. On the other hand, SVAR showed that *bai-1 *and *tsp-1 *genes are induced by the *p53 *gene product in Hela cells. It is known that the *bai-1 *gene codes for a member of the secretin receptor family, which contains at least one functional p53-binding site within an intron, and its product is postulated to be an inhibitor of angiogenesis and a tumor growth suppressor [40]. Similarly, the *tsp-1 *gene codes for an adhesive glycoprotein that mediates cell-to-cell and cell-to-matrix interactions and has been shown to play a role in platelet aggregation, angiogenesis, and tumorigenesis [41]. Taken together, the p53-mediated upregulation of *bai-1 *and *tsp-1 *genes may be a mechanism to evade cell migration and angiogenesis, features which are commonly absent in Hela cells. We noticed that the classical p53 targets, such as *gadd45 *and *p21*, do not appear to be directly regulated by p53 in the SVAR analysis (Figure 5). This may be explained by the fact that the time-series length is not large enough. It is important to note that our previous study applying DVAR (Dynamic Vector AutoRegressive) [42], it was possible to identify these connectivities.

The observed p53-independent transcriptional regulation of the *p21 *gene (Figure 5), appears to be unrelated to cell cycle arrest, as discussed below.

The STAT3 protein is a member of the STAT protein family. In response to cytokines and growth factors, it forms both homo- or heterodimers with other STAT proteins and the complex translocates to the nucleus, where they act as transcriptional activators. STATs mediate the cell response to different stimuli, playing a key role in several cellular processes, such as cell growth and apoptosis [43]. As shown, using the SVAR method (Figure 5), STAT3 regulates the expression of the cycle positive regulator Cyclin D1 and of the anti-apoptotic protein Bcl-2. It has already been reported that constitutive activation of STAT 3 correlates with *cyclin d1 *and *bcl-2 *gene overexpression, thus providing a novel prognostic marker for head and neck squamous cell carcinoma [44]. Moreover, repression of *p53 *gene expression by STAT3 is likely to have an important role in development of tumors [45]. These evidence point to an involvement of STAT3 in cell cycle progression and transformation of Hela cells.

Our *in silico *analysis also highlighted an unexpected behavior for the *p21 *gene, independently of p53 regulation. This alternative regulation has already been described for other cell types [46], but still remains unclear in the case of Hela cells. Although p21 is not a transcription factor, it is conceivable that indirect effects of p21 on cellular gene expression of well-known cell cycle progression promoters, such as Cyclin D1 and apoptosis inhibitors, such as Bcl-2 may mediate some unexpected functions in Hela cells. These functions appear to be unrelated to growth inhibition and cell cycle arrest, supporting the hypothesis that p53-independent regulation of p21 could be one of the signaling pathways activated during tumorogenesis and/or tumor progression in Hela cells as well as in other cancer types [47,48]. Future efforts directed to evaluate this hypothesis include gene transfection of p21 mutants lacking the p53 and STAT3-binding sites and subsequent, analysis of the newly identified p21 targets gene expression and changes in Hela cells phenotype and tumorigenicity.

It is interesting that, even using a small dataset, the SVAR method allowed identification of actual regulations, as detailed above, illustrating the power of this technique. In general, the methods reported in the literature are not based on a statistical test due to difficulties generated by the fact that the number of samples is lower than the number of parameters to be estimated, consequently, they do not provide an objective control for false-positives.

The main advantage of the sparse vector autoregressive model (SVAR), compared with other connectivity models, is that it models a Granger-causal network with a number of genes that is larger than the number of samples, in other words, it is useful to model "large" networks with a statistical test for each one of the edges. To the best of our knowledge, the approach taken here is the only one that combines these two advantages since other methods which model "large" networks usually do not present statistical tests for the edges. Moreover, "large" gene-gene networks are commonly dealt with in pairwise comparisons. Using SVAR, it is possible to infer partial Granger-causalities resulting in a lower number of spurious edges than pairwise comparisons.

Since SVAR deals with the multivariate case, the definition of Granger causality becomes complex, because of the existence of multi-steps connectivities. In the present report, identification of Granger causality using the SVAR model is related to the definition of partial Granger-causality given by [49]. By definition of Granger's causality [49] the SVAR model allows analysis of cycles containing networks. Therefore, there is no *a priori *assumption that the network must be a DAG (Directed Acyclic Graph), as assumed by other methods [5,9]. As a consequence, the SVAR method can be used to model networks with cycles. This is of extreme importance, since it is well-known that genetic regulatory networks maintain their control and balance by a number of positive/negative feedback cycles.

There is a class of Bayesian network with MCMC algorithm which may integrate expression data with multiple sources of information [8]. The advantages of integrating multiple sources of information, i.e., adding *a priori *knowledge, is speculative. Integration of *a priori *knowledge maybe interesting to recover more realistic connections and to increase the power of the test. However, it also lead to a bias depending on the kind of information assumed in the model. In this actual stage of development of SVAR, integration of different information is not possible since only gene expression levels are used to estimate Granger causality. Further studies may be focused on integrating biological information to improve the power of SVAR.

The experimental comparison between SVAR and other methods is difficult since SVAR is the only one which has a statistical test for gene-gene networks comprising a notion of Granger-causality. The Graphical Gaussian Models reported by Schäfer and Strimmer, which apply partial correlations in the context of (*n > m*) is the closest one to SVAR, presenting a statistical test, however, the edges obtained by this approach represent instantaneous associations (correlations), failing to provide a notion of Granger-causality, i.e., the edges have no direction.

Differently from score functions, which pose difficult interpretations or subjective choices of the threshold to determine where there is (or not) an edge, a statistical test is an objective way to determine whether there is an edge and what is the rate of type I error.

In this work, we considered only lags of first order, but it is relatively straightforward to generalize this method to analyze SVAR models with orders higher than one. However, this issue depends on the number of parameters to be estimated and the time series length.

The complexity of the proposed inference is linear to the number of genes, since only one regression is performed for each gene.

There are other approaches for variable selection based on stepwise methods. Unfortunately, these methods are not consistent when *n > m *[50], i.e., even increasing the sample size (*T *→ ∞), there is no guarantee that the set of non-zero coefficients is the correct one. This result does not change even if all subsets of variables are explored.

In contrast to LASSO, one may choose to use other penalized regressions, such as the more popular Ridge [51] or the non-negative Garrote [52]. Ridge does not set the variables to zero, resulting in models with difficult interpretations. Comparing LASSO to non-negative Garrote, the latter is worse than LASSO when multicolinearity is present in the data [23]. Therefore, LASSO seems to be the most appropriate in identifying gene regulatory networks.

Another advantage of SVAR is the fact that it does not require model pre-specification; therefore, this method is unbiased and makes it possible to infer new connections, not just quantifying the dependence level measured by already known edges. Furthermore, it is not necessary to discretize gene expression values to Boolean variables, as in the Boolean network models [17]; therefore, there is no loss of information.

In the SVAR approach, to render the application of statistics when (*n > m*) feasible, we used the fact that the metabolic networks are sparsely connected as part of the solution. Therefore, the number of variables to be analyzed decreases significantly, resulting only in variables whose estimated coefficients are large enough to be tested and rejected as being different from zero.

## Conclusion

In summary, here we introduce the SVAR method to model gene regulatory networks in the present context, where the number of samples is often lower than the number of genes. With this method, it is possible to naturally model networks with feedback loops and to infer partial Granger causalities without any *a priori *information, which minimizes the number of spurious causalities. Moreover, we present a statistical test to control for the false discovery rate, a task which was not previously possible in several other proposed gene regulatory network models.

## Methods

Firstly, we describe the classical vector autoregressive model (VAR) and, then, we explore the feasibility of using LASSO regression as part of a technique for variable selection, by introducing the sparse vector autoregressive model (SVAR). The statistical test for the edges is also presented followed by the control of the false positives. To simplify the description of these methods, we describe both the SVAR and the VAR of order one, but they could easily be generalized to higher orders. After this description, we present the algorithm to construct artificial regulatory networks based on scale-free topology, since metabolic networks were described to have power-law distributions in the nodes' degrees [25]. We use this artificial network to evaluate the performance of our proposed model. Finally, the SVAR model is applied to actual biological data.

### Statistical background

Granger (1969) [53] defined a concept of causality, which is easy to deal with in the context of VAR models; therefore, it has become quite popular in recent years [54]. The idea is that a cause cannot come after the effect. Thus, in the case of VAR(1) (VAR of order one) [54], if a gene *i *at time (*t *- 1) affects another gene *j *at time *t*, the former should help to predict the target gene expression.

A first order VAR model is described as shown:

*y*_*t *_= *A*_1_*y*_*t*-1 _+ *ε*_*t *_*t *= 2,..., *T*

where *T *is the time-series' length (number of microarrays) *y*_*t *_is an *n *× 1 vector of gene expression (where *n *is the number of genes), the normally distributed disturbance *ε*_*t *_is an *n *× 1 vector with mean zero and covariance matrix Ω, and *A*_1 _is an *n *× *n *matrix of parameters (connectivities). The disturbances *ε*_*t *_are serially uncorrelated, but may be contemporaneously correlated. Thus E(εtε′t)
 MathType@MTEF@5@5@+=feaafiart1ev1aaatCvAUfKttLearuWrP9MDH5MBPbIqV92AaeXatLxBI9gBaebbnrfifHhDYfgasaacH8akY=wiFfYdH8Gipec8Eeeu0xXdbba9frFj0=OqFfea0dXdd9vqai=hGuQ8kuc9pgc9s8qqaq=dirpe0xb9q8qiLsFr0=vr0=vr0dc8meaabaqaciaacaGaaeqabaqabeGadaaakeaacqWGfbqrcqGGOaakiiGacqWF1oqzdaWgaaWcbaGaemiDaqhabeaakiqb=v7aLzaafaWaaSbaaSqaaiabdsha0bqabaGccqGGPaqkaaa@361B@ = Ω, where Ω is an *n *× *n *matrix. It is important to highlight that, in this multivariate model, each gene may depend not only on its own past values, but, also, on the past values of the other genes. Thus if *y*_*it *_denotes the *i*th element in *y*_*t*_, the *i*th row yields

*y*_*it *_= *a*_*i*1_*y*_1,*t*-1 _+ *a*_*i*2_*y*_2,*t*-1 _+ ...+ *a*_*iN*_*y*_*N*,*t*-1 _+ *ε*_*it*_, *i *= 1,...,*n*

This model can be estimated by Ordinary Least Squares (OLS), simply by regressing each variable on the lags of itself and the other variables.

Therefore, we can re-write it as

*Z *= *Xβ *+ *EE*_*i *_~ *N*(0, Ω) *i *= 1,..., *n*

where *E*_*i *_follows a multivariate Gaussian distribution *N*(0, Ω), with zero mean 0_(*n*×1) _and covariance matrix Ω.

We define *m *= *T *- 1 and introduce the notation:

*Z*_(*m×n*) _= [*y*_2_,...,*y*_*t*_,...,*y*_*T*_]' = [*z*_1_,...,*z*_*i*_,...,*z*_*n*_],

β(n×n)=A′1=[β1,...,βn],
 MathType@MTEF@5@5@+=feaafiart1ev1aaatCvAUfKttLearuWrP9MDH5MBPbIqV92AaeXatLxBI9gBaebbnrfifHhDYfgasaacH8akY=wiFfYdH8Gipec8Eeeu0xXdbba9frFj0=OqFfea0dXdd9vqai=hGuQ8kuc9pgc9s8qqaq=dirpe0xb9q8qiLsFr0=vr0=vr0dc8meaabaqaciaacaGaaeqabaqabeGadaaakeaaiiGacqWFYoGydaWgaaWcbaGaeiikaGIaemOBa4Maey41aqRaemOBa4MaeiykaKcabeaakiabg2da9iqbdgeabzaafaWaaSbaaSqaaiabigdaXaqabaGccqGH9aqpcqGGBbWwcqWFYoGydaWgaaWcbaGaeGymaedabeaakiabcYcaSiabc6caUiabc6caUiabc6caUiabcYcaSiab=j7aInaaBaaaleaacqWGUbGBaeqaaOGaeiyxa0LaeiilaWcaaa@472B@

*X*_(*m×n*) _= [*y*_1_...*y*_*m*_]',

*E*_(*m×n*) _= [*ε*_2_,...,*ε*_*t*_,...,*ε*_*T*_]'

The explicit solution of the OLS estimator is

*β *= (*X'X*)^-1^*X'Z*

Therefore, one can carry out separate regression analyses for each gene. In other words, it is possible to separately estimate each column *β*_*i *_of *β*:

β^i=(X′X)−1X′zii=1,...,n
 MathType@MTEF@5@5@+=feaafiart1ev1aaatCvAUfKttLearuWrP9MDH5MBPbIqV92AaeXatLxBI9gBaebbnrfifHhDYfgasaacH8akY=wiFfYdH8Gipec8Eeeu0xXdbba9frFj0=OqFfea0dXdd9vqai=hGuQ8kuc9pgc9s8qqaq=dirpe0xb9q8qiLsFr0=vr0=vr0dc8meaabaqaciaacaGaaeqabaqabeGadaaakeaafaqabeqacaaabaacciGaf8NSdiMbaKaadaWgaaWcbaGaemyAaKgabeaakiabg2da9iabcIcaOiqbdIfayzaafaGaemiwaGLaeiykaKYaaWbaaSqabeaacqGHsislcqaIXaqmaaGccuWGybawgaqbaiabdQha6naaBaaaleaacqWGPbqAaeqaaaGcbaGaemyAaKMaeyypa0JaeGymaeJaeiilaWIaeiOla4IaeiOla4IaeiOla4IaeiilaWIaemOBa4gaaaaa@44C1@

where *z*_*i *_is the *i*-th column of *Z*.

In order to specify the distribution of the *j*-th element of β^
 MathType@MTEF@5@5@+=feaafiart1ev1aaatCvAUfKttLearuWrP9MDH5MBPbIqV92AaeXatLxBI9gBaebbnrfifHhDYfgasaacH8akY=wiFfYdH8Gipec8Eeeu0xXdbba9frFj0=OqFfea0dXdd9vqai=hGuQ8kuc9pgc9s8qqaq=dirpe0xb9q8qiLsFr0=vr0=vr0dc8meaabaqaciaacaGaaeqabaqabeGadaaakeaaiiGacuWFYoGygaqcaaaa@2E64@, let us denote the *j*-th diagonal element of (*X'X*)^-1 ^by *w*_*jj*_. Then, we may assert the statistical test as

β^ijσ^2wjj∼t(m−n)i=1,...,n
 MathType@MTEF@5@5@+=feaafiart1ev1aaatCvAUfKttLearuWrP9MDH5MBPbIqV92AaeXatLxBI9gBaebbnrfifHhDYfgasaacH8akY=wiFfYdH8Gipec8Eeeu0xXdbba9frFj0=OqFfea0dXdd9vqai=hGuQ8kuc9pgc9s8qqaq=dirpe0xb9q8qiLsFr0=vr0=vr0dc8meaabaqaciaacaGaaeqabaqabeGadaaakeaafaqabeqacaaabaWaaSaaaeaaiiGacuWFYoGygaqcamaaBaaaleaacqWGPbqAcqWGQbGAaeqaaaGcbaWaaOaaaeaacuWFdpWCgaqcamaaCaaaleqabaGaeGOmaidaaOGaem4DaC3aaSbaaSqaaiabdQgaQjabdQgaQbqabaaabeaaaaGccqWI8iIocqWG0baDcqGGOaakcqWGTbqBcqGHsislcqWGUbGBcqGGPaqkaeaacqWGPbqAcqGH9aqpcqaIXaqmcqGGSaalcqGGUaGlcqGGUaGlcqGGUaGlcqGGSaalcqWGUbGBaaaaaa@4A00@

under the null hypothesis, where *t*(*m *- *n*) denotes a *t *distribution of (*m *- *n*) degrees of freedom and

σ^2=1m−n(Z−Xβ^)′(Z−Xβ^)=E′Em−n
 MathType@MTEF@5@5@+=feaafiart1ev1aaatCvAUfKttLearuWrP9MDH5MBPbIqV92AaeXatLxBI9gBaebbnrfifHhDYfgasaacH8akY=wiFfYdH8Gipec8Eeeu0xXdbba9frFj0=OqFfea0dXdd9vqai=hGuQ8kuc9pgc9s8qqaq=dirpe0xb9q8qiLsFr0=vr0=vr0dc8meaabaqaciaacaGaaeqabaqabeGadaaakeaaiiGacuWFdpWCgaqcamaaCaaaleqabaGaeGOmaidaaOGaeyypa0ZaaSaaaeaacqaIXaqmaeaacqWGTbqBcqGHsislcqWGUbGBaaGaeiikaGIaemOwaOLaeyOeI0IaemiwaGLaf8NSdiMbaKaacuGGPaqkgaqbaiabcIcaOiabdQfaAjabgkHiTiabdIfayjqb=j7aIzaajaGaeiykaKIaeyypa0ZaaSaaaeaacuWGfbqrgaqbaiabdweafbqaaiabd2gaTjabgkHiTiabd6gaUbaaaaa@49F5@

It is to point out that these definitions will work only if *m *> *n*. Additionally, it is also well known that OLS does not ensure sparse connectivity patterns for *A*.

To overcome these problems, in the next section, we introduce the sparse vector autoregressive model.

### Sparse Vector AutoRegressive (SVAR)

Consider *Z*, *β*, *X *and *E *as described above.

According to [55-58], the LASSO (Least Absolute Shrinkage and Selection Operator) regression [23] can be carried out by iterative application of:

β^ik+1=(X′X+λ2D(β^ik))−1X′zii=1,...,n and k=1,...,Nit
 MathType@MTEF@5@5@+=feaafiart1ev1aaatCvAUfKttLearuWrP9MDH5MBPbIqV92AaeXatLxBI9gBaebbnrfifHhDYfgasaacH8akY=wiFfYdH8Gipec8Eeeu0xXdbba9frFj0=OqFfea0dXdd9vqai=hGuQ8kuc9pgc9s8qqaq=dirpe0xb9q8qiLsFr0=vr0=vr0dc8meaabaqaciaacaGaaeqabaqabeGadaaakeaafaqabeqacaaabaacciGaf8NSdiMbaKaadaqhaaWcbaGaemyAaKgabaGaem4AaSMaey4kaSIaeGymaedaaOGaeyypa0JaeiikaGIafmiwaGLbauaacqWGybawcqGHRaWkcqWF7oaBdaahaaWcbeqaaiabikdaYaaakiabdseaejabcIcaOiqb=j7aIzaajaWaa0baaSqaaiabdMgaPbqaaiabdUgaRbaakiabcMcaPiabcMcaPmaaCaaaleqabaGaeyOeI0IaeGymaedaaOGafmiwaGLbauaacqWG6bGEdaWgaaWcbaGaemyAaKgabeaaaOqaaiabdMgaPjabg2da9iabigdaXiabcYcaSiabc6caUiabc6caUiabc6caUiabcYcaSiabd6gaUjabbccaGiabbggaHjabb6gaUjabbsgaKjabbccaGiabdUgaRjabg2da9iabigdaXiabcYcaSiabc6caUiabc6caUiabc6caUiabcYcaSiabd6eaonaaBaaaleaacqWGPbqAcqWG0baDaeqaaaaaaaa@6474@

where *N*_*it *_is the number of iterations (we set *N*_*it *_= 30 to our analysis), *λ *is the regularization parameter which determines the amount of penalization enforced, D(β^ik)
 MathType@MTEF@5@5@+=feaafiart1ev1aaatCvAUfKttLearuWrP9MDH5MBPbIqV92AaeXatLxBI9gBaebbnrfifHhDYfgasaacH8akY=wiFfYdH8Gipec8Eeeu0xXdbba9frFj0=OqFfea0dXdd9vqai=hGuQ8kuc9pgc9s8qqaq=dirpe0xb9q8qiLsFr0=vr0=vr0dc8meaabaqaciaacaGaaeqabaqabeGadaaakeaacqWGebarcqGGOaakiiGacuWFYoGygaqcamaaDaaaleaacqWGPbqAaeaacqWGRbWAaaGccqGGPaqkaaa@3418@ is a diagonal matrix defined by

D(θ)=diag(p′λ(θ)/θ)k=1,...,n
 MathType@MTEF@5@5@+=feaafiart1ev1aaatCvAUfKttLearuWrP9MDH5MBPbIqV92AaeXatLxBI9gBaebbnrfifHhDYfgasaacH8akY=wiFfYdH8Gipec8Eeeu0xXdbba9frFj0=OqFfea0dXdd9vqai=hGuQ8kuc9pgc9s8qqaq=dirpe0xb9q8qiLsFr0=vr0=vr0dc8meaabaqaciaacaGaaeqabaqabeGadaaakeaafaqabeqacaaabaGaemiraqKaeiikaGccciGae8hUdeNaeiykaKIaeyypa0JaemizaqMaemyAaKMaemyyaeMaem4zaCMaeiikaGIafmiCaaNbauaadaWgaaWcbaGae83UdWgabeaakiabcIcaOiab=H7aXjabcMcaPiabc+caViab=H7aXjabcMcaPaqaaiabdUgaRjabg2da9iabigdaXiabcYcaSiabc6caUiabc6caUiabc6caUiabcYcaSiabd6gaUbaaaaa@4BB9@

and

p′λ(θ)=λsign(θ)
 MathType@MTEF@5@5@+=feaafiart1ev1aaatCvAUfKttLearuWrP9MDH5MBPbIqV92AaeXatLxBI9gBaebbnrfifHhDYfgasaacH8akY=wiFfYdH8Gipec8Eeeu0xXdbba9frFj0=OqFfea0dXdd9vqai=hGuQ8kuc9pgc9s8qqaq=dirpe0xb9q8qiLsFr0=vr0=vr0dc8meaabaqaciaacaGaaeqabaqabeGadaaakeaacuWGWbaCgaqbamaaBaaaleaaiiGacqWF7oaBaeqaaOGaeiikaGIae8hUdeNaeiykaKIaeyypa0Jae83UdWMaem4CamNaemyAaKMaem4zaCMaemOBa4MaeiikaGIae8hUdeNaeiykaKcaaa@3F13@

At each iteration, the regression coefficients of each gene with all others are weighted according to their current size and several coefficients are successively down-weighted and set to zero.

The covariance matrix of the estimators may then be approximated by:

(X′X+λ2D(β^))−1X′X(X′X+λ2D(β^))−1σ^2
 MathType@MTEF@5@5@+=feaafiart1ev1aaatCvAUfKttLearuWrP9MDH5MBPbIqV92AaeXatLxBI9gBaebbnrfifHhDYfgasaacH8akY=wiFfYdH8Gipec8Eeeu0xXdbba9frFj0=OqFfea0dXdd9vqai=hGuQ8kuc9pgc9s8qqaq=dirpe0xb9q8qiLsFr0=vr0=vr0dc8meaabaqaciaacaGaaeqabaqabeGadaaakeaacqGGOaakcuWGybawgaqbaiabdIfayjabgUcaRGGaciab=T7aSnaaCaaaleqabaGaeGOmaidaaOGaemiraqKaeiikaGIaf8NSdiMbaKaacqGGPaqkcqGGPaqkdaahaaWcbeqaaiabgkHiTiabigdaXaaakiqbdIfayzaafaGaemiwaGLaeiikaGIafmiwaGLbauaacqWGybawcqGHRaWkcqWF7oaBdaahaaWcbeqaaiabikdaYaaakiabdseaejabcIcaOiqb=j7aIzaajaGaeiykaKIaeiykaKYaaWbaaSqabeaacqGHsislcqaIXaqmaaGccuWFdpWCgaqcamaaCaaaleqabaGaeGOmaidaaaaa@4EFD@

where *σ*^2 ^is an estimate of the error variance

σ^2=1m−n−c(Z−Xβ^)′(Z−Xβ^)=E′Em−n−c
 MathType@MTEF@5@5@+=feaafiart1ev1aaatCvAUfKttLearuWrP9MDH5MBPbIqV92AaeXatLxBI9gBaebbnrfifHhDYfgasaacH8akY=wiFfYdH8Gipec8Eeeu0xXdbba9frFj0=OqFfea0dXdd9vqai=hGuQ8kuc9pgc9s8qqaq=dirpe0xb9q8qiLsFr0=vr0=vr0dc8meaabaqaciaacaGaaeqabaqabeGadaaakeaaiiGacuWFdpWCgaqcamaaCaaaleqabaGaeGOmaidaaOGaeyypa0ZaaSaaaeaacqaIXaqmaeaacqWGTbqBcqGHsislcqWGUbGBcqGHsislcqWGJbWyaaGaeiikaGIaemOwaOLaeyOeI0IaemiwaGLaf8NSdiMbaKaacuGGPaqkgaqbaiabcIcaOiabdQfaAjabgkHiTiabdIfayjqb=j7aIzaajaGaeiykaKIaeyypa0ZaaSaaaeaacuWGfbqrgaqbaiabdweafbqaaiabd2gaTjabgkHiTiabd6gaUjabgkHiTiabdogaJbaaaaa@4E6D@

and *c *is the number of variables *β *set to zero by LASSO regression.

When σ^2
 MathType@MTEF@5@5@+=feaafiart1ev1aaatCvAUfKttLearuWrP9MDH5MBPbIqV92AaeXatLxBI9gBaebbnrfifHhDYfgasaacH8akY=wiFfYdH8Gipec8Eeeu0xXdbba9frFj0=OqFfea0dXdd9vqai=hGuQ8kuc9pgc9s8qqaq=dirpe0xb9q8qiLsFr0=vr0=vr0dc8meaabaqaciaacaGaaeqabaqabeGadaaakeaaiiGacuWFdpWCgaqcamaaCaaaleqabaGaeGOmaidaaaaa@2FA5@ replaces *σ*^2^, we get the result that the statistical test is

β^σ^2wjj~t(m−n−c)i=1,...,n
 MathType@MTEF@5@5@+=feaafiart1ev1aaatCvAUfKttLearuWrP9MDH5MBPbIqV92AaeXatLxBI9gBaebbnrfifHhDYfgasaacH8akY=wiFfYdH8Gipec8Eeeu0xXdbba9frFj0=OqFfea0dXdd9vqai=hGuQ8kuc9pgc9s8qqaq=dirpe0xb9q8qiLsFr0=vr0=vr0dc8meaabaqaciaacaGaaeqabaqabeGadaaakeaafaqabeqacaaabaWaaSaaaeaaiiGacuWFYoGygaqcaaqaamaakaaabaGaf83WdmNbaKaadaahaaWcbeqaaiabikdaYaaakiabdEha3naaBaaaleaacqWGQbGAcqWGQbGAaeqaaaqabaaaaOGaeiOFa4NaemiDaqNaeiikaGIaemyBa0MaeyOeI0IaemOBa4MaeyOeI0Iaem4yamMaeiykaKcabaGaemyAaKMaeyypa0JaeGymaeJaeiilaWIaeiOla4IaeiOla4IaeiOla4IaeiilaWIaemOBa4gaaaaa@49A9@

under the null hypothesis, where *t*(*m *- *n *- *c*) denotes a *t *distribution of (*m *- *n *- *c*) degrees of freedom and *w*_*jj *_is the *j*-th diagonal element of

(X′X+λ2D(β^))−1X′X(X′X+λ2D(β^))−1
 MathType@MTEF@5@5@+=feaafiart1ev1aaatCvAUfKttLearuWrP9MDH5MBPbIqV92AaeXatLxBI9gBaebbnrfifHhDYfgasaacH8akY=wiFfYdH8Gipec8Eeeu0xXdbba9frFj0=OqFfea0dXdd9vqai=hGuQ8kuc9pgc9s8qqaq=dirpe0xb9q8qiLsFr0=vr0=vr0dc8meaabaqaciaacaGaaeqabaqabeGadaaakeaacqGGOaakcuWGybawgaqbaiabdIfayjabgUcaRGGaciab=T7aSnaaCaaaleqabaGaeGOmaidaaOGaemiraqKaeiikaGIaf8NSdiMbaKaacqGGPaqkcqGGPaqkdaahaaWcbeqaaiabgkHiTiabigdaXaaakiqbdIfayzaafaGaemiwaGLaeiikaGIafmiwaGLbauaacqWGybawcqGHRaWkcqWF7oaBdaahaaWcbeqaaiabikdaYaaakiabdseaejabcIcaOiqb=j7aIzaajaGaeiykaKIaeiykaKYaaWbaaSqabeaacqGHsislcqaIXaqmaaaaaa@4C06@

It is important to emphasize that the number of variables set to zero in this method will depend on the value of the regularization parameter *λ*, with higher values implying on the selection of fewer variables.

In our work, the value of the tuning parameter *λ *was selected as the value that minimizes the generalized cross validation criterion (GCV).

Let *q*(*λ*) = *tr*{*X*(*X'X *+ *λ*^2^*D*(*β*))^-1^*X'*} and *rss*(*λ*) be the residual sum of squares for the constrained fit with constraint *λ*, the generalized cross-validation statistic can be written as:

GCV=1mrss(λ){1−q(λ)/m}2
 MathType@MTEF@5@5@+=feaafiart1ev1aaatCvAUfKttLearuWrP9MDH5MBPbIqV92AaeXatLxBI9gBaebbnrfifHhDYfgasaacH8akY=wiFfYdH8Gipec8Eeeu0xXdbba9frFj0=OqFfea0dXdd9vqai=hGuQ8kuc9pgc9s8qqaq=dirpe0xb9q8qiLsFr0=vr0=vr0dc8meaabaqaciaacaGaaeqabaqabeGadaaakeaacqWGhbWrcqWGdbWqcqWGwbGvcqGH9aqpdaWcaaqaaiabigdaXaqaaiabd2gaTbaadaWcaaqaaiabdkhaYjabdohaZjabdohaZjabcIcaOGGaciab=T7aSjabcMcaPaqaaiabcUha7jabigdaXiabgkHiTiabdghaXjabcIcaOiab=T7aSjabcMcaPiabc+caViabd2gaTjabc2ha9naaCaaaleqabaGaeGOmaidaaaaaaaa@4849@

The minimum value for GCV was achieved by the L-BFGS-B algorithm [59], which was implemented in the function *optim *of the R statistical environment.

For more details on the statistical properties of LASSO in autoregressive models see [60].

### Controlling the number of false-positives

To control the type I error in cases of multiple tests of hundreds of edges, we applied the FDR method [24].

Firstly, assume that of the *n *hypotheses tested {H10,H20,...,Hn0}
 MathType@MTEF@5@5@+=feaafiart1ev1aaatCvAUfKttLearuWrP9MDH5MBPbIqV92AaeXatLxBI9gBaebbnrfifHhDYfgasaacH8akY=wiFfYdH8Gipec8Eeeu0xXdbba9frFj0=OqFfea0dXdd9vqai=hGuQ8kuc9pgc9s8qqaq=dirpe0xb9q8qiLsFr0=vr0=vr0dc8meaabaqaciaacaGaaeqabaqabeGadaaakeaacqGG7bWEcqWGibasdaqhaaWcbaGaeGymaedabaGaeGimaadaaOGaeiilaWIaemisaG0aa0baaSqaaiabikdaYaqaaiabicdaWaaakiabcYcaSiabc6caUiabc6caUiabc6caUiabcYcaSiabdIeainaaDaaaleaacqWGUbGBaeaacqaIWaamaaGccqGG9bqFaaa@3EF9@, where Hj0
 MathType@MTEF@5@5@+=feaafiart1ev1aaatCvAUfKttLearuWrP9MDH5MBPbIqV92AaeXatLxBI9gBaebbnrfifHhDYfgasaacH8akY=wiFfYdH8Gipec8Eeeu0xXdbba9frFj0=OqFfea0dXdd9vqai=hGuQ8kuc9pgc9s8qqaq=dirpe0xb9q8qiLsFr0=vr0=vr0dc8meaabaqaciaacaGaaeqabaqabeGadaaakeaacqWGibasdaqhaaWcbaGaemOAaOgabaGaeGimaadaaaaa@303D@ is the null hypothesis of the *j*-th test and {*p*(1), *p*(2),...,*p*(*n*)} their corresponding p-values, *n*_0 _are the number of true null hypotheses and the other (*n *- *n*_0_) hypotheses are false.

Let *p*(1) ≤ *p*(2) ≤ ... ≤ *p*(*n*) be the ordered observed p-values of each test. Define

l−max{i:p(i)≤inq}
 MathType@MTEF@5@5@+=feaafiart1ev1aaatCvAUfKttLearuWrP9MDH5MBPbIqV92AaeXatLxBI9gBaebbnrfifHhDYfgasaacH8akY=wiFfYdH8Gipec8Eeeu0xXdbba9frFj0=OqFfea0dXdd9vqai=hGuQ8kuc9pgc9s8qqaq=dirpe0xb9q8qiLsFr0=vr0=vr0dc8meaabaqaciaacaGaaeqabaqabeGadaaakeaacqWGSbaBcqGHsislcqWGTbqBcqWGHbqycqWG4baEcqGG7bWEcqWGPbqAcqGG6aGocqWGWbaCcqGGOaakcqWGPbqAcqGGPaqkcqGHKjYOdaWcaaqaaiabdMgaPbqaaiabd6gaUbaacqWGXbqCcqGG9bqFaaa@42DE@

and reject H(1)0...H(l)0
 MathType@MTEF@5@5@+=feaafiart1ev1aaatCvAUfKttLearuWrP9MDH5MBPbIqV92AaeXatLxBI9gBaebbnrfifHhDYfgasaacH8akY=wiFfYdH8Gipec8Eeeu0xXdbba9frFj0=OqFfea0dXdd9vqai=hGuQ8kuc9pgc9s8qqaq=dirpe0xb9q8qiLsFr0=vr0=vr0dc8meaabaqaciaacaGaaeqabaqabeGadaaakeaacqWGibasdaqhaaWcbaGaeiikaGIaeGymaeJaeiykaKcabaGaeGimaadaaOGaeiOla4IaeiOla4IaeiOla4IaemisaG0aa0baaSqaaiabcIcaOiabdYgaSjabcMcaPaqaaiabicdaWaaaaaa@397F@. If no such *i *exists, reject all null hypothesis.

FDR is defined as the expected proportion (*q*) of incorrectly rejected null hypotheses (type I error) in a list of all rejected hypotheses.

### Artificial regulatory networks

The description that many networks in nature have a power-law degree distribution was first addressed by [61]. In their random graph model, called scale-free graph, it is described how these networks grow and expand, being based on two generic mechanisms, which are common to several networks in the real world. Several networks in the real world start from a small number of nodes and grow by continuous addition of new nodes, therefore, the number of nodes increases throughout the lifetime of the network. When a new node is added to the network, its attachment is preferential, i.e., the probability of a new node connects to the existing nodes is not uniform as in a random graph [62]. There is a higher probability to be linked to a node that already has a large number of connections, resulting in a power-law degree distribution. In other words, the probability *P*(*v*) that a node in the network is connected to *v *other nodes decays as a power-law. Therefore, the degree distribution has a power-law tail *P*(*v*) ~ *v*^-*γ*^, where *γ *is a scalar which represents the rate of decayment of the degree distribution. In our case, the nodes are representing the genes and the connections are the Granger-causal relationships.

This scale-free graph can be constructed as below:

1. Growth: Starting with a small number *z*_0 _of genes, at each iteration, a new gene with *z *≤ (*z*_0_) edges are added. This new gene is connected to the genes already present in the network with a preferential attachment.

2. Preferential attachment: The gene with which the new gene will connect is selected in a non-deterministic fashion. Assume that the probability *π *that a new gene will be connected to gene *i *depends on the degree *d*_*i *_of that gene which is already in the network. Therefore:

π(di)=di∑jdj
 MathType@MTEF@5@5@+=feaafiart1ev1aaatCvAUfKttLearuWrP9MDH5MBPbIqV92AaeXatLxBI9gBaebbnrfifHhDYfgasaacH8akY=wiFfYdH8Gipec8Eeeu0xXdbba9frFj0=OqFfea0dXdd9vqai=hGuQ8kuc9pgc9s8qqaq=dirpe0xb9q8qiLsFr0=vr0=vr0dc8meaabaqaciaacaGaaeqabaqabeGadaaakeaaiiGacqWFapaCcqGGOaakcqWGKbazdaWgaaWcbaGaemyAaKgabeaakiabcMcaPiabg2da9maalaaabaGaemizaq2aaSbaaSqaaiabdMgaPbqabaaakeaadaaeqaqaaiabdsgaKnaaBaaaleaacqWGQbGAaeqaaaqaaiabdQgaQbqab0GaeyyeIuoaaaaaaa@3D0B@

Since we are interested in causal relationships, we need to define a direction for each edge. Therefore, there is a third step in our graph construction. In our simulations, the probability attributed to add an edge from *i *to *j *is the same from *j *to *i*, i.e., 0.5.

After *T*_*step *_iterations, the constructed random scale-free like network is composed of *n *= *T*_*step *_+ *z*_0 _genes and z * *T*_*step *_+ *z*_*edges *_Granger-causal relationships, where *z*_*edges *_is the initial number of edges.

The graph constructed using the algorithm described above may be represented by its adjacency matrix *A*, i.e., where there is an edge from gene *i *to gene *j *it was set to *A*[*i, j*] = 0.8, and 0 otherwise, in our simulations. This adjacency matrix *A *corresponds to the matrix *A *described in equation 1. The time-series' lag was set to one in our simulations, therefore, set *m *= *T *- 1.

To construct the corresponding time-series for each gene, firstly, generate normally distributed random numbers with zero mean and unit variance for each gene *i *= 1,...,*n *for the time step *t *= 1, *y*_*i*1 _= *ε*_*i*_. Then, use equation 2 to generate the time-series for each gene *i *= 1,...,*n*, time step *t *= 2,...,*T*.

### Implementation

We implemented our program using R [63], a statistical computing environment. Computation was conducted under a Pentium IV CPU 3.06 GHz, 2.5 GB of RAM.

### Application to real data

We applied the SVAR approach to HeLa cell cycle gene expression data collected by Whitfield *et al*. (2002) [64]. Gene expression was measured using microarrays manufactured in the Stanford Microarray Facility. The data used contain 48 time points distributed at one hour intervals with one reading at each time point, synchronized by double thymidine block (described as Experiment 3 in the web page [65]). The 94 genes were selected from actual biological microarray data on the basis of there association with cell cycle regulation and tumor development. The HeLa cell cycle lasts 16 hours. These data were downloaded from: [65].

## Authors' contributions

AF has made substantial contributions to the conception and design of the study, analysis and interpretation of data. JRS has made substantial contributions to the analysis and interpretation of mathematical results. HMGM has made substantial contributions to the analysis and interpretation of biological data. AF, JRS and HMGM have been involved in drafting of the manuscript. RY and SM have discussed the mathematical results. MCS has discussed the biological results. CEF has directed the work. RY, SM, MCS and CEF critically revised the manuscript for important intellectual content. All authors read and approved the final manuscript.
